# Optimizing collaborative learning in online courses

**DOI:** 10.1111/tct.13243

**Published:** 2020-09-09

**Authors:** Jascha de Nooijer, Francine Schneider, Daniëlle ML Verstegen

**Affiliations:** ^1^ School of Health Professions Education (SHE) Department of Health Promotion Maastricht University Maastricht the Netherlands; ^2^ Care and Public Health Research Institute Department of Health Promotion Maastricht University Maastricht the Netherlands

## Abstract

Currently, higher education institutes are urged to adapt their education programmes rapidly to online courses. This toolbox article provides recommendations for optimising collaborative learning in online courses from the perspective of course design, and the roles of teachers and students, all illustrated in our example. With regards to course design, it is recommended to construct learning tasks for which students need to collaborate to reach a shared goal, use collaboration scripts to structure activities and communication, manage expectations about collaboration, provide room for discussion about the team process, facilitate autonomy and use existing communication tools. The presence of teachers online is essential, to provide feedback on the content and to guide team processes. Finally, students are recommended to get to know fellow students, to create a positive atmosphere and to reflect on the collaboration. We conclude that online collaborative learning can work well, but requires a balance between course structure and autonomy, and needs active monitoring during implementation. If this is done, it is perfectly possible to engage students and teachers, to support deep learning and to develop collaboration skills.

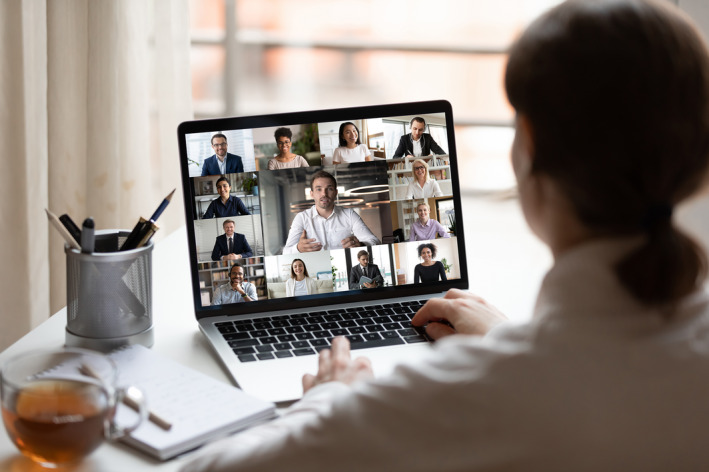

## INTRODUCTION

In this toolbox article we provide recommendations for optimising collaboration in online courses. Not only as a result of but triggered more urgently by the worldwide COVID‐19 crisis, higher education institutes are urged to adapt their education provision rapidly from face‐to‐face teaching to an online mode. Needless to say, this is a huge challenge for programmes traditionally delivered on campus, such as (under)graduate education in health professions, including medical curricula. Such programmes often use problem‐based learning (PBL), following the philosophy that learning occurs when it is constructive, contextual, collaborative and self‐directed.^1^ But how do we offer online courses without losing these principles?

Learners in health professions education are mostly adults and often combine work and study. Given the benefits of collaborative learning for academic success, there is a need to develop learning activities online that facilitate this process. Collaboration stimulates deep learning.^1^ Moreover, students who need to collaborate develop skills that are so important for the labour market.^2^, ^3^ Furthermore, collaborative learning online enhances the opportunities for intercultural and interprofessional learning, which is often difficult to organise in person.

The combination of online learning and collaboration is by no means self‐evident, however. Collaboration requires technical facilities and skills to use online collaboration tools.^4^ Furthermore, group dynamics and the development of trust are different in virtual teams.^5^, ^6^

The purpose of this toolbox article is to provide recommendations for the design and the execution of online courses to optimise collaborative learning from the perspective of course design, and the roles of teachers and students, as illustrated by our example (Box 1). The lessons learned here may help others to optimise collaboration in their own online courses. Box 2 presents a summary of the steps taken to apply the recommendations.

Box 1 **The online course on Intervention Development at Maastricht University, the Netherlands**

This 8‐week course is part of a regular master’s degree programme in Health Education and Promotion. The study load is 20 hours per week. Students earn six European Credit Transfer System (ECTS) credits upon completion. Start and end dates are fixed. The general aim is to outline and justify a health promotion intervention. Related to communication and collaboration, two specific learning outcomes were defined: (i) students can critically discuss their own and other’s opinions, ideas and work; and (ii) students effectively cooperate in small groups with persons of different backgrounds and initial levels. Participants are full‐time or part‐time students with a diversity of backgrounds. Groups of four or five students work online on a group assignment, where they have to design a health promotion intervention based on the intervention mapping (IM) protocol. No face‐to‐face meetings were scheduled. The group work was assessed and includes the paper, a justification of the choices made related to the task, and a justification of how the group cooperated and how each individual member contributed. Furthermore, via an individual exam, the student’s understanding of and application of the protocol was assessed.

During the development and implementation phases, the course took problem‐based learning principles into account.^1^ This means that learners work with authentic problems, construct new knowledge on the basis of prior knowledge and collaborate with other participants, while monitoring their own learning process.

Box 2 **Recommendations applied to Intervention Development**

1. Students work in small groups on an outline and justification for the development of an intervention (self‐selected health problem). This task is too big for one student to accomplish alone. It requires the division of work and frequent discussion, as each step of intervention mapping (IM) builds on the previous steps, including the logic model of the problem, the programme outcomes and objectives, programme design, programme production, implementation plan and evaluation plan.
2. The collaboration script corresponds with the various steps of IM that students are learning about. It points to when and where students must contribute themselves, as well as read and provide feedback on others’ contributions.
3. Students explicitly agree on how to collaborate using a Team Charter, a written document that specifies the roles, content and deadlines of the task. Therefore, students write down in advance when and where the group members meet, and what each group member has to do before that meeting. Afterwards, they describe whether or not everybody was present and finished the work that had to be done for that meeting.
4. Students use the synchronous (mostly Skype) and asynchronous (e.g. e‐mail, discussion board in the learning environment) tools that they are familiar with for various communication and collaboration purposes.
5. Teachers respond quickly so as not to disturb the flow of the process. Teachers provide frequent feedback, encourage participation, and show respect and care for others.
6. Teachers record short video clips of general feedback for all groups on a weekly basis and give specific written feedback on the submitted assignments, to facilitate frequent (informal) interaction on issues related to the content, to gain a sense of whether students are heading in the right direction and whether they have understood the assignment correctly.
7. Students introduce themselves in detail by completing a profile and recording a video. Students are explicitly invited to watch and respond to their fellow students’ clips beforehand.
8. The team process is discussed every week using the tasks explicitly defined in the Team Charter. Therefore, students also write down in advance what piece of information each group member contributes and when this should be distributed among the group members. Afterwards, they describe whether or not this was completed in time, and if not, who was responsible and why. The group is asked to provide two tops (things that went well) and two tips (areas for improvement) on collaboration as a team.
9. Several facets contribute to creating a positive atmosphere:
  - motivated students who are willing to invest in collaboration online;
  - the group has the autonomy to arrange the assignment according to their own preferences, i.e. they have the freedom to decide when they do (part of) a task within a timeslot of a week, their approach and which tools to use;
  - weekly reflection sessions on the task and group process, including tips and tops; and
  - motivated teachers who are focused on the task and the group process.

## COURSE DESIGN

### Recommendation 1: construct learning tasks that force students to collaborate in order to attain a common goal

Teamwork or working in small groups succeeds when people share a common goal and are interdependent.^7^ When people have a shared goal, they need to interact and connect with others. This ‘relatedness’ is identified as one of the three basic needs people have that enhance intrinsic motivation and growth.^8^ Interdependency contributes to relatedness because it encourages interaction and provides an opportunity to connect with others.^9^

When people have a shared goal, they need to interact and connect with others

### Recommendation 2: develop collaboration scripts that explicitly structure activities and communication

Tasks should be clearly described in the online collaborative learning environment, not only in terms of the expected end product but also in terms of the necessary steps to be taken. A collaboration script is a set of instructions prescribing how students should form groups, how they should interact and collaborate, and how they should solve the problem. Task‐oriented scripts can be seen as a helpful form of scaffolding because they structure the work and clarify what is expected from students and what they should do when.^10^

Task‐oriented scripts can be seen as a helpful form of scaffolding because they structure the work and clarify what is expected from students and what they should do when

### Recommendation 3: organise discussions about team processes and make expectations explicit

Online group work requires attention for task division, setting clear expectations and discussing timelines. This requires having organised discussions about team processes, including how to reach an agreement on how and when to communicate. This rarely happens spontaneously in virtual teams.^6^ Thus, it is important to incorporate it in the collaboration script. An ‘explicit didactic contract’ between teachers and students, and among students themselves, is required in order to regulate the mode of collaboration.^10^ As a result of limited ‘real‐life’ contact and the lack of nonverbal cues in online communication, both teachers and students should make their expectations explicit to avoid misunderstandings. Global instructions often result in implicit expectations that are harder to verify in the online learning environment, compared with the face‐to‐face situation.^10^

### Recommendation 4: provide a range of communication and collaboration tools

Different learning situations require appropriate tools, and teams should be encouraged to select the tools that suit their needs. It helps when the institution facilitates the availability of these tools. Software tools can enable effective synchronous (e.g. skype, zoom, google hangouts and blackboard collaborate) and asynchronous (e.g. e‐mail, discussion boards, blogs, Wikis and podcasts) collaboration. Synchronous communication can be used to optimise the alignment of ideas, to make important decisions or to solve conflicts, and asynchronous communication can be used when topics need further elaboration. All are necessary to engage learners and to facilitate deep learning.^1^

## THE ROLE OF THE TEACHER

### Recommendation 5: be present in the online environment

In online learning, the role of the teacher influences students’ satisfaction, learning and persistence.^11^ Garrison distinguishes three types of presence: cognitive presence (the process of inquiry that contributes to meaningful learning, e.g. providing feedback); teaching presence (developing and implementing the course, e.g. encouraging participation); and social presence (aimed at creating an atmosphere of trust and belonging, e.g. showing respect and care for others).^12^ For both teachers and students, however, being present is a point of concern in the online context, because of the characteristics of asynchronous communication and potential disconnectedness.

for both teachers and students being present is a point of concern in the online context, because of the characteristics of asynchronous communication and potential disconnectedness

### Recommendation 6: provide feedback on task and group processes

Feedback is the main source of interaction between teachers and students. Positive, constructive feedback enhances competence.^8^, ^9^ Online, feedback becomes even more important.^12^ Providing feedback is a form of cognitive presence and is essential for learning, although this is often postponed until assignments are officially handed in.^12^ Elaborate formative feedback during the course is useful, however, as it allows students to directly apply what they learn from the feedback in their final assignment. Teachers should pay attention to task‐related aspects and to interpersonal collaboration in order to facilitate personal growth.^8^ Collaboration online comes with similar challenges as face‐to‐face collaboration, such as personal clashes and conflict; however, online communication poses some extra difficulties, including technical barriers and the lack of nonverbal information. Particularly in asynchronous communication, it is harder to intervene immediately and effectively.^5^, ^13^ Monitoring group processes should be built intentionally into the design of the course, but it is also a continuous process during course implementation. If necessary, teachers should act to correct students who are out of line, obviously in a respectful manner.^12^, ^13^, ^14^

## THE ROLE OF THE PARTICIPANT

### Recommendation 7: invest in getting to know each other in order to become a group that collaborates effectively

Groups develop in several stages,^15^ and the participants’ willingness to interact and connect permits growth.^8^ Taking time to get to know each other is therefore essential for ‘warming up’, and is vital in the online setting because nonverbal communication is mostly absent and there is far less occasion for (informal) contact.^5^ When students and teachers never meet in person it is important to plan a moment to ‘virtually’ meet each other, preferably at the start of a synchronous session. Virtual introductions should focus first on interpersonal interaction between the group members and not immediately on task work.

Taking time to get to know each other is therefore essential for ‘warming up’, and is vital in the online setting …

### Recommendation 8: reflect on collaboration

Facilitating and monitoring the group process is the responsibility of teachers and students. In online settings it is sometimes less obvious when this should happen. It is important for group members to be explicit on the agreements about collaboration, however, including specific roles, like the chair or note taker, and aspects that need to be improved. If a Team Charter (Box 2) is used, one way to do this is to regularly review this document. ‘Free‐riding’ (benefiting from the course without expending effort) occurs often in collaborative learning.^14^ It is important to discuss how the team wants to deal with free‐riders, and what the team is willing to accept. On the other hand, it may be important to accept that not everyone in the team can always contribute equally, especially when students are working professionals coming from highly diverse backgrounds.

### Recommendation 9: put effort into creating a positive team atmosphere

Group work in online learning seems to focus on rich and relevant discourse related to the task, and less on personal relationships. As students are not present in person, frequent interaction is needed to enhance personal identity and projection within a team. Although immediacy behaviours (e.g. greetings, addressing group mates by name) and interactive indicators (e.g. approval, agreement, invitations) support the development of online discussions, such behaviours seem to become less important, whereas interactive indicators seem to grow in importance, over time. Interactive indicators show real attendance and commitment to the group. Verstegen found that virtual teams who put effort into maintaining a positive atmosphere function better.^6^ An inspection of team communication showed various ways of maintaining positivity, e.g. explicitly giving compliments, asking others personally for their input, offering help with technology and showing understanding for those who could not always contribute.

## CONCLUSION

Collaboration in online courses in a PBL context is feasible when several perspectives are considered (design of the course, the role of teachers and the role of students). The online collaboration seems to work well, mainly related to task aspects. Maintaining a balance between course structure (content and collaboration) and autonomy requires a delicate touch and a well‐thought‐out design. During implementation, the aspects of collaboration need constant monitoring and responding to, if necessary. If this is done, it becomes perfectly possible to engage students and teachers, to support deep learning and to develop collaboration skills using tools that have now become increasingly available in higher education.
